# Hyaluronan-binding system for sperm selection enhances pregnancy rates in ICSI cycles associated with male factor infertility

**DOI:** 10.5935/1518-0557.20170002

**Published:** 2017

**Authors:** Renata F Erberelli, Renato M Salgado, Dirceu H Mendes Pereira, Philip Wolff

**Affiliations:** 1 Clínica Genics - Medicina Reprodutiva e Genômica, São Paulo - Brazil

**Keywords:** ICSI, Spermatozoa, Male Infertility, Hyaluronic acid

## Abstract

**Objectives:**

The aim of the present study was to compare two procedures for sperm selection in ICSI cycles - conventional morphology sperm selection (ICSI-PVP) and chemical selection through Hyaluronan-treated petri dishes (PICSI), when male factor was associated.

**Methods:**

The evaluated parameters were semen quality, fertilization and cleavage rates, chemical and clinical pregnancy rates, as well as abortion rate. Fifty-six ICSI cycles were included in this report, 19 cycles using PICSI and 37 using conventional ICSI.

**Results:**

PICSI and ICSI showed, respectively, the following outcome: fertilization rates 71.93% (123/171) and 64.14% (127/198); cleavage rates 95.12% (117/123) and 95.27% (121/127); chemical pregnancy rates 63.15% (12/19) and 27.03% (10/37); clinical pregnancy rates 42.10% (8/19) and 16.21% (6/37); and abortion rates 33.33% (4/12) and 40.00% (4/10). According to both Fisher's Exact Test and Chi-square Test, chemical pregnancy (*p* = 0.05) and clinical pregnancy (*p* = 0.09) rates were significantly higher in the PICSI group. *p* values ≤ 0.05 were consider statistically significant.

**Conclusions:**

The present data indicates that ICSI cycles that used the PICSI technique had a considerably higher chance (≈5 fold) to achieve pregnancy than those who had sperm selected only by morphology assessment. Teratozoospermic patients were those who benefited most with PICSI. Therefore, the technique should be included in laboratory routine with low cost, avoiding the selection of immature sperm with increased rates of peroxidation and DNA fragmentation. Prospective and randomized studies should be applied to strengthen this suggestion.

## INTRODUCTION

Sperm quality has direct influence in fertilization and posterior embryonic development. Sperm with normal morphology selected for injection into the oocyte, according to the proposed criteria by the World Health Organization, show higher pregnancy rates in ICSI cycles, and have a high prediction value for therapeutic techniques (Berkovitz *et al*., 2005).

Male infertility factors are present in 35% of couples with infertility issues (Madera, 2002; Speroff & Fritz, 2005), and the causes are related to conditions such as obesity, diabetes, hypertension, infections, genetic problems and/or external factors like stress, smoking, drug abuse and others. Damage in the acrosome and sperm plasma membrane or even in the chromatin may lead to impaired embryo development, failure to implant in the uterus and recurrent miscarriages (Seli *et al*., 2004; Aitken *et al*., 2014).

The main consequence of changes in sperm patterns is the increase in the Reactive Oxygen Species (ROS) (Noblanc *et al*., 2014). ROS excessive production is related to severe defects in sperm features, like severe *oligozoospermia* and possibly the accumulation of cytoplasm in the middle piece (Twigg *et al*., 1998).

The differentiation of sperm is called spermiogenesis, the stage in which the sperm forms the acrosome, condenses the chromatin through protamination (nuclear compaction) and rearrangement of organelles (Perreault *et al*., 1988; Hess & de Franca, 2008). It is an important stage in the development of CD44 receptors for Hyaluronan, present in the granulosa layer of the cumulus-oocyte complex, as well as receptors that bind the Zona Pellucida proteins ZP1, ZP2 and ZP3, which are fundamental in the fertilization process. High rates of sperm DNA fragmentation are directed related to men with altered patterns of semen parameters (Irvine *et al*., 2000). Viable techniques for the selection of possible sperm with low fragmentation rates in real time were adopted and those have been used in *in vitro* (IVF) fertilization processes, such as the selection technique by Hyaluronan (HA) in sperm binding, and the morphologic selection technique in high magnification sperm (IMSI-MSOME) (Bartoov *et al.*, 2002).

Oocytes are naturally encircled by a layer of cells of the *cumulus oophorus*-*corona radiate* complex, constituted by an extracellular matrix of polymerized HA and glycoproteins, and it is in this region that the first interactions between the sperm and the oocyte occur. Sperm can fail to interact with the oocyte due to the lack of membrane remodeling in the last stage of the spermiogenesis (Hess & de Franca, 2008; Huszar *et al.,* 2000).

Two proteins are related to sperm maturity: the augmented expression of the thermic shock protein 2 (HSPA2), which impairs nuclear and cytoplasmic maturation (Huszar *et al*., 2000) and the reduced expression of kinase creatine (KC), which is involved only in the maturation of the cytoplasm (Cayli *et al*., 2003). Sperm considered 'immature' present deficiencies in the ZP and HA binding sites (Parmegiani *et al.,* 2012). This binding can be artificially induced *in vitro* by two ready-to-use mechanisms: *1)* Drops of HA exposed in the bottom of a plastic culture dish (PICSI^®^); *2)* Viscous medium that contains HA (Sperm Slow - Medicult Origio).

The work of Huszar *et al*. (2003) showed that sperm that bind to the HA system are mature, which can be attested through immunohistochemical like kinase creatine (KC) and the HSPA2 protein for the analysis of the cytoplasm extrusion, by aniline blue Staining for the detection of permanent histones and *pisum sativum agglutinin* for the detection of acrossomal integrity, as well as by the *Fertilight Test* technique for the detection of sperm viability.

The aim of the present study was to carry out a comparative analysis between two groups: Hyaluronan-binding system for sperm selection for ICSI procedures and another group of conventional morphology sperm selection (ICSI-PVP), both in cycles with only male infertility factor. The evaluated parameters were semen quality, fertilization and cleavage rates, chemical and clinical pregnancy, and miscarriage rates.

## METHODS

### Experimental Design

Fifty-six ICSI cycles were analyzed. After anamnesis and clinical-laboratorial diagnoses, only couples with moderate to severe male factor were chosen for the study, according to the WHO (2010) criteria. Cycles that used PESA, TESA or microtese procedures were excluded from the study. The cycles were carried out in the *Genics* – *Medicina Reprodutiva e Genômica* Clinic, São Paulo, Brazil, from July 2013 to July 2014. Patients were allocated into two experimental groups. Out of those 56 cycles, 19 cycles of ICSI were carried out through the HA PICSI^®^ system and posterior morphology (PICSI Group), and 37 cycles were carried out only through the conventional selection system (PVP Group), according to considering the parameters described in the WHO Manual (2010).

The male factors were analyzed in relation to sperm quality and divided into eight categories: moderate *oligozoospermia*, severe *Oligozoospermia*, moderate *Oligo-astenozoospermia*, severe *Oligo-asthenozoospermia*, *Teratozoospermia*, *Necro-teratozoospermia, Asthenozoospermia and Hypospermia.* The reference limits for those categories follow specific values determined by the World Health Organization (2010) (Table 1).

**Table 1:** The reference by the World Health Organization (2010).

Category	Reference Value
Moderate Oligozoospermia	Sperm Concentration: 5-10 million/ml
Severe Oligozoospermia	Sperm Concentration: 5-10 million/ml
Moderate Oligoasthenozoospermia	Sperm concentration: 5-10 million/ml and Progressive motility (PR) < 32%
Severe Oligoasthenozoospermia	Sperm concentration: < 5 million/ml and Progressive motility (PR) < 32%
Teratozoospermia	Sperm morphology: normal < 4%
Necro-teratozoospermia	Sperm vitality: < 58% and Sperm morphology: normal < 4%
Asthenozoospermia	Progressive motility (PR) < 32%
Hypospermia	Sperm volume < 1.5 ml

Concerning the PICSI group the average age of the female patient was 35.05 ± 3.67, with a median of 12 aspirated oocytes per patient. As for the ICSI-PVP group, the average age of the women female patient was 35.76 ± 3.44, with a median of 6 aspirated oocytes per patient.

### Sperm Preparation

After semen liquefaction (30 minutes at 37.0°C), macroscopic and microscopic analyses were carried out following the procedures established by the Clinic and the samples were prepared for sperm selection with G-MOPS Plus buffered medium (Vitrolife - Göteborg, Sweden), using the techniques of discontinuous gradient (Isolate Lower and Upper, Irvine Scientific – Santa Ana, CA), Swim-up or Sperm-wash, depending on concentration and motility of each sample.

### Selection of Sperm through the Hyaluronan (HA)-binding Technique

Every sample used through this technique should have at least one million progressive motile (PR) sperm per ml of semen after the processing of the sample, as this selection technique is based on motility patterns. A 50 mm x 9 mm sterile petri dish was used for the selection of "mature" sperm (PICSI^®^ MidAtlantic Diagnoses - Origio, Denmark). The dish was prepared two hours before its use to hydrate the drops of HA exposed at the bottom of the dish.

For the HA hydration 5µl-droplets of G-Mops Plus were used, which were covered with 3 ml of mineral oil (Irvine Scientific – Santa Ana, CA) and kept at room temperature for 2 hours. An average of 1µl of processed sperm sample was carefully and laterally added to the HA hydrated droplets, and kept at 36.8°C for at least 5 minutes, to allow the sperm to bind to the glycosaminoglycan. The spermatozoa attached by the head to the dish on the region exposed to HA and that vigorously wiggled their tail were transferred to a droplet of PVP with 10% of HSA (Irvine Scientific - Santa Ana, CA) for morphologic selection, according to the standards previously proposed by the World Health Organization (2010). After this selection, each sperm was immobilized and aspirated by the tail through a micropipette for the oocyte injection, in a 5µl droplet of G-Mops Plus.

### Embryo Culture in Sequential Media and Embryo Transfer (ET)

After the sperm injection, all oocytes were individually placed in 30µl droplets of G_1_ plus (Vitrolife - Göteborg, Sweden) and kept at 36.8°C in a conventional incubator (6% CO_2_). Every 16 or 18 hours the oocytes were checked for fertilization and classified regarding the formation of normal zygote. For cleavage and embryo development, these pre-embryos were washed and kept in low O_2_ *Trigas* incubators (6%CO_2_-5%O_2_-89%N) (Minc Benchtop Cook Medical) in G_1_ plus medium. Over the following days cleavage and embryo quality were analyzed. The pre-embryos were taken to an extended culture to the blastocyst stage, and were transferred from on D3 to G_2_ plus (Sequential culture system) (Vitrolife - Göteborg, Sweden).

βeta-HCG was measured from the 10th day to the 13th day after ET, in order to confirm chemical pregnancy. Ultrasound exams were carried out from the 6^th^ to the 8^th^ week for the confirmation of pregnancy through the visualization of a gestational sac (SG) and fetal heartbeat.

### Statistical Analysis

Statistical analyses were performed using Chi-square Test (*x*^2^) was applied to analyze differences in fertilization, cleavage and chemical pregnancy rates between both groups, and Fisher's Exact Test was applied for clinical pregnancy and abortion outcomes, where values of *p* ≤ 0.05 were considered statistically significant. The *x*^2^ Test was also used for comparison of prevalence among the male factor categories. Concerning the female patients' age variables among the groups we used the Kolmogorov-Smirnov Test to confirm normal distribution in both groups. The non-parametric Mann-Whitney Test was used for the variables of the numbers of aspirated follicles between groups.

## RESULTS

A total of 56 ICSI cycles were included in this study. Nineteen ICSI cycles were carried out with sperm selected by the PICSI system and 37 cycles were carried out only by sperm selection using conventional morphological and motility assessment.

Normal distribution was observed in both groups for the female patients age, which shows that there is no significant difference in average age between both groups (*p* = 0.48). The average number of aspirated oocytes for the PICSI group was significantly higher when compared to the conventional group PVP (*p* = 0.02). The table 2 shows the distribution of categories of associated male factor between the two analyzed groups.

**Table 2:** The distribution of categories of associated male factor between Conventional PVP and PISCI groups.

	Conventional PVP (37 - 100%)	PISCI (HA Binding) (19 - 100%)
Moderate Oligozoospermia	3/3 (8.1%)	3/19 (15.8%)
Severe Oligozoospermia	1/37 (2.7%)	1/19 (5.3%)
Moderate Oligo-asthenozoospermia	2/37 (5.4%)	0/19 (0.0%)
Severe Oligo -asthenozoospermia	3/37 (8.1%)	1/19 (5.3%)
Teratozoospermia	19/37 (51.4%)	12/19 (63.2%)
Necro -teratozoospermia	1/37 (2.7%)	0/19 (0.0%)
Asthenozoospermia	6/37 (16.2%)	1/19 (5.3%)
Hypospermia	2/37 (5.4%)	1/19 (5.3%)

When we associated the βeta-HCG results from both groups with the categories of semen alteration we observed a significant difference only in Teratozoospermia (*p* = 0.03), as shown in the table 3.

**Table 3:** The βeta-HCG results with the categories of semen alteration. Comparison between Conventional PVP and PISCI groups.

	Conventional PVP (9)	PICSI (HA Binding) (12)	*P* - *Value*
Moderate Oligozoospermia	1/3 (33.3%)	2/3 (66.6%)	1.00
Severe Oligozoospermia	0/1 (0.0%)	0/1 (0.0%)	1.00
Moderate Oligo- asthenozoospermia	1/2 (50.0%)	0/0 (-)	1.00
Severe Oligo-asthenozoospermia	0/3 (0.0%)	1/1 (100.0%)	1.00
Teratozoospermia	6/19 (31.6%)	9/12 (75.0%)	0.03
Necro- teratozoospermia	0/1 (0.0%)	0/0 (-)	1.00
Asthenozoospermia	0/6 (0.0%)	0/1 (0.0%)	1.00
Hypospermia	1/2 (50.0%)	0/1 (0.0%)	1.00

Chemical pregnancy and clinical pregnancy rates were significantly higher in the PICSI group, with respective values of *p* = 0.05 and *p* = 0.009; however, there was no significant difference in abortion rate (Table 4).

**Table 4:** Conventional PVP x PISCI groups. Laboratory and Clinical results.

Rates	PICSI(HA Binding)	Conventional PVP	*p-value*
Fertilization	123/171 (71.93%)	127/198 (64.14%)	0.11
Cleavage	117/123 (95.12%)	121/127 (95.27%)	0.96
Chemical pregnancy	12/19 (63.15%)	10/37 (27.03%)	0.05
Clinical pregnancy	8/19 (42.10%)	6/37 (16.21%)	0.009
Abortion	4/12 (33.33%)	4/10 (40.00%)	1.00

## DISCUSSION

Considering that both groups included only cases with male factor infertility and that the number of transferred embryos was similar in both groups (average: 2.52 embryos/TE in the PICSI Group and 2.13 embryos/TE in the ICSI-PVP Group), the outcome of the present study is solid and shows that the HA (PICSI) selection system is efficient, as this group of patients had a higher clinical pregnancy rate when compared to the patients from the control group (conventional PVP) (*p* < 0.05). The statistical analysis showed that the ones who used the PICSI technique had 4.62 more chances of becoming pregnant. The abortion rates tended to be lower in this group, but it was not statistically significant, probably due to the low number of patients included in this study. All clinical pregnancies were successful, resulting in live healthy new-born babies without malformations.

We found that the number of aspirated oocytes in the PICSI group was higher in comparison to the PVP conventional group. Ovarian stimulating cycles that did not follow a specific protocol could explain those discrepant values, which might lead to a conspicuous variation on the number of recovered oocytes, even if there was no variation on the age of the female patients.

We also observed that the patients who had *teratozoospermia,* as an infertility male factor, could benefit from the PICSI technique more than other factors associated with sperm changes, suggesting the therapeutic use of this procedure for all cases with aberrant sperm morphology.

In a study by Twigg *et al.* (1998) sperm with genetic defects could form normal pro-nuclei after *in vitro* fertilization with posterior cleavage, generating embryos with aneuploidy - which normally results in pregnancy loss. Moreover, embryos derived from aberrant sperm present high levels of fragmentation during cleavage and development. Spermatozoa selected by the HA-binding technique have better internal and external structures (Prinosilova *et al.,* 2009; Parmegiani *et al.,* 2010a, b), and shows a reduction in the risk of aneuploidy or presenting fragmented DNA (Parmegiani *et al.,* 2012). The compaction of the sperm chromatin provides protection and maintenance of DNA integrity, making it more resistant to mutations and environmental stress. The ruptures of these bridges can cause nuclear de-compaction and de-condensation (Perreault *et al.,* 1988).

Defects in the sperm DNA-protein complex can be analyzed by simple diagnostic techniques, to obtain further data on the reproductive health of the man, with the use of DNA fragmentation tests by TUNEL, chromatin dispersion (SCD), flow cytometry or through staining with toluidine blue and acridine orange (Tejada *et al*., 1984), among others. These tests are not always used for the indication of adequate treatment, as the semen samples collected from the same man show discrepant semen patterns in different collections (WHO., 2010).

Bartoov *et al*. (2002) developed a method called MSOME (*motile sperm organelle morphology examination*), that selects sperm for ICSI using high magnification (> 6000×) in real time, managing to get a better sperm morphologic evaluation, mainly the analysis of the presence of vacuoles in the head (location, size and numbers), aiding in the selection of sperm with better mitochondrial function, intact chromatin, as well as reducing the number of embryos with aneuploidy (Parmegiani *et al.,* 2012). The technique has been successfully used for oligo-asthenoteratozoospermic patients (Kim *et al*., 2014) and for couples that had successive implantation failures, reducing clinic abortion rates by 50% (Oliveira *et al.,* 2011). The downside of this technique is that, apart from the high costs, it may take 60-120 minutes from the selection of the sperm to its injection, depending on semen quality. This period may affect the cells, as the sperm may vacuolize its nuclei after 2 hours in warm medium (Peer *et al*., 2007; Parmegiani *et al.,* 2012).

Based on the present and previous data, we may infer that sperm selected by the HA technique before ICSI offer better predictive value to form competent embryos, thus helping to optimize treatment results (Balaban *et al.,* 2003). In culture media, HA does not negatively affect embryo development (Van Den Bergh *et al.,* 2009), and can be metabolized by the oocyte (Balaban *et al*., 2003; Van Den Bergh *et al.,* 2009).

Apparently, the benefits of the HA binding system for sperm selection shows significant positive results only for cycles with associated male factor. For instance, in the study by Majumdar & Majumdar (2013), the morphologic selection and the HA system techniques were compared in relation to patients with infertility without apparent reason and without associated male factors. Indeed, the results did not show significant differences, but demonstrated a tendency in the reduction of abortion rates in the cases that used the HA system for sperm selection.

In conclusion, the HA binding system for sperm selection can be used within the laboratory routine and can mimic a "physiologic" choice of the male gamete at low costs, increasing pregnancy rates and reducing possible genetic complications. Robust prospective randomized studies must be carried out to strengthen this hypothesis.
